# An epidemiological perspective of personalized medicine: the Estonian experience

**DOI:** 10.1111/joim.12320

**Published:** 2015-01-26

**Authors:** L Milani, L Leitsalu, A Metspalu

**Affiliations:** 1 Estonian Genome Center, University of TartuTartu, Estonia; 2 Institute of Molecular and Cell Biology, University of TartuTartu, Estonia

**Keywords:** biobanks, eHealth, electronic medical records, Estonia, genomic medicine, personalized medicine

## Abstract

Milani L, Leitsalu L, Metspalu A (University of Tartu). An epidemiological perspective of personalized medicine: the Estonian experience (Review). J Intern Med 2015; 277: 188–200.

The Estonian Biobank and several other biobanks established over a decade ago are now starting to yield valuable longitudinal follow-up data for large numbers of individuals. These samples have been used in hundreds of different genome-wide association studies, resulting in the identification of reliable disease-associated variants. The focus of genomic research has started to shift from identifying genetic and nongenetic risk factors associated with common complex diseases to understanding the underlying mechanisms of the diseases and suggesting novel targets for therapy. However, translation of findings from genomic research into medical practice is still lagging, mainly due to insufficient evidence of clinical validity and utility. In this review, we examine the different elements required for the implementation of personalized medicine based on genomic information. First, biobanks and genome centres are required and have been established for the high-throughput genomic screening of large numbers of samples. Secondly, the combination of susceptibility alleles into polygenic risk scores has improved risk prediction of cardiovascular disease, breast cancer and several other diseases. Finally, national health information systems are being developed internationally, to combine data from electronic medical records from different sources, and also to gradually incorporate genomic information. We focus on the experience in Estonia, one of several countries with national goals towards more personalized health care based on genomic information, where the unique combination of elements required to accomplish this goal are already in place.

## Introduction

Personalized medicine is an area that is receiving increasingly more attention internationally [1]. It is defined as medical practice that utilizes personal medical information, including health behaviour, traditional medical test results, symptoms, family history, environmental factors and genomic information, to implement new routines for diagnosing and treating diseases [2]. The main benefit of a more personalized approach is the expected shift of medical practices towards preventive health care, based on targeted screening programmes combined with early intervention and treatment, instead of reactive treatment of disease. As such, its implementation is dependent on robust known risk factors, electronic medical records (EMRs), automated decision support systems and specific training of relevant personnel. A more personalized approach towards health care will engage individuals more actively in their health management, which is a prerequisite for improving public health.

**Figure 1:**
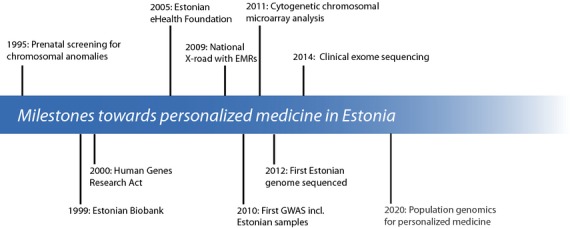
Major milestones in medical genetics (top) and genomics (bottom) in Estonia. The years shown mark the official launch, establishment or publication of the ‘milestone’, although preliminary work was initiated much earlier. EMR, electronic medical record; GWAS, genome-wide association study.

The power of biobanks for epidemiological research was recognized over a decade ago in Estonia (Fig.1). Furthermore, Estonia was, together with Iceland, amongst the first to initiate a population-based biobank designed to use biomarkers combined with medical history and lifestyle information in the study of common diseases and traits [3]. In contrast to the Estonian Biobank, which is maintained by the government, the Icelandic initiative has always been a private enterprise, with all samples and data initially owned by deCODE genetics, Inc. and recently acquired by Amgen, Inc. Prior to the establishment of the Estonian Biobank, the Estonian Human Genes Research Act was passed by the Parliament of Estonia [4]. According to this law, the Estonian Biobank has the right to collect, store and use biological samples and phenotype information for genetic research and is further expected to use the results to improve public health. The necessary infrastructure to promote secure electronic exchange of medical data, a nationwide technical infrastructure (termed the X-road platform), is already established and maintained by the state [5]. As of 2010, medical data from hospitals, primary care physicians and pharmacies (digital prescription records) are all accessible through the X-road in a strictly regulated manner. Because of the existence of all the elements mentioned above, Estonia is in a globally unique position for the implementation of genomic medicine on a national scale.

Although the potential of genomic medicine has long been recognized [6], genomic data are still not incorporated in clinical decision-making for common diseases. The clinical validity and utility of the genomic disease variants identified so far are still being questioned. The aim of this review is to provide a summary of the genomic variants that are associated with common diseases and their power in combined genetic risk scores, and discuss the possibilities of using genetic data in health care. For illustration, we use examples from studies of type 2 diabetes (T2D), coronary artery disease (CAD) and breast cancer, and focus on the experience in Estonia where the unique combination of the key elements for nationwide implementation of personalized medicine are already in place.

## Biobanks for epidemiological research

Research and development in the field of biomedicine is highly dependent on the availability of biological samples and associated epidemiological data. The Biobanking and Biomolecular Resources Research Infrastructure (BBMRI) is one of the largest research infrastructure projects in Europe with more than 225 associated organizations from over 30 countries combined into a 54-member consortium [7,8]. BBMRI is now implemented under the European Research Infrastructure Consortium (ERIC), a legal framework that functions as a platform to create opportunities for long-term cooperation between the members of the consortium [9]. The Public Population Project in Genomics and Society (P^3^G) is another international consortium dedicated to providing a multidisciplinary infrastructure for unified health and social research conducted around the world [10,11]. These and other global consortia [12] enable the international research community to develop more effective strategies for the implementation of modern healthcare efforts aimed at disease prevention, tailored treatments and the promotion of the long-term health of individuals in a harmonized manner.

Estonia was amongst the founding members of the P^3^G and BBMRI-ERIC. The Estonian Biobank, similar to several other biobanks, is a volunteer-based sample of the adult population, with close to 52 000 participants representing 5% of the population (age ≥18 years). All participants have undergone a standardized health examination, donated blood samples for purification of DNA, white blood cells and plasma, and completed a questionnaire on health-related topics, such as lifestyle, diet and clinical diagnoses [13]. A significant proportion of the cohort (*n* = 20 000) has been genotyped using genome-wide single nucleotide polymorphism (SNP) arrays, and data from nuclear magnetic resonance (NMR) spectroscopy of blood plasma are available for 12 000 participants. The metabolic parameters determined by NMR spectroscopy include low molecular weight compounds (*n* = 24), lipoproteins (*n* = 91) and lipids (*n* = 25) [14]. All participants have signed a broad informed consent form, which allows the continual updating of epidemiological data through periodic linking to national electronic databases and registries.

The Estonian Genome Center (EGCUT) is a research institute of the University of Tartu that manages and maintains the Estonian Biobank. As a result of the combination of a vast amount of both genotype and phenotype data, the EGCUT participates in several consortia focusing on large-scale genome-wide association studies (GWASs), including Genetic Investigation of ANthropometric Traits (GIANT), Cohorts for Heart and Aging Research in Genomic Epidemiology (CHARGE), European Network for Genetic and Genomic Epidemiology (ENGAGE), DIAbetes Genetics Replication And Meta-analysis (DIAGRAM), Meta-Analyses of Glucose and Insulin-related traits (MAGIC), Coronary ARtery DIsease Genome-wide Replication And Meta-analysis (CARDIOGRAM) and Metabolites And GeNETIcs Consortium (MAGENETIC).

## Genomics of common diseases

The parallel progress of the HapMap project [15], generating a list of tag SNPs that capture most of the common variation in the human genome, and development of dense microarrays that allow the simultaneous genotyping of hundreds of thousands of SNPs has facilitated the large-scale performance of GWASs using samples from cases and controls based on phenotype data available in different biobanks. The success of 5 years of discoveries from GWASs has recently been summarized by Visscher *et al*. [16]. To date, SNPs in or near 7100 different genes have been reported to be associated with over 1000 different traits; this is summarized in the GWASs catalogue available online [17,18]. Table1 shows the most recent results from a selection of highly successful GWASs, with associated loci explaining between 2% and 60% of the heritability of the studied traits. The molecular mechanisms through which the associated SNPs act are still unknown for a large proportion of them, mostly due to extended regions of strong correlations between SNPs. However, the associations are robust and different studies are taking place to provide evidence for biological processes that link the associated variants to the studied phenotypes.

**Table 1:** Proportion of disease or trait heritability explained by GWAS hits^a^

Disease/trait	Number of associated loci	Heritability explained by associated loci (%)	Reference
Type 2 diabetes	76	∼10	75,76
BMI	36	∼10	77
Lipids	157	∼30	27,78
Breast cancer	67	∼14	45
Height	180	∼10	79
Type 1 diabetes	40	∼60	80,81
Rheumatoid arthritis	48	∼51	82,83
Inflammatory bowel disease	163	∼14	84
Schizophrenia	108	∼3–7	85
Bipolar disorder	56	∼2	86

GWAS, genome-wide association study; BMI, body mass index.

a Adapted and updated from Visscher *et al*. [16].

Using data generated by the ENCODE project [19], which integrates multiple types of functional data, Schaub *et al*. [20] were able to identify putative functional annotations for up to 80% of all investigated SNP–trait associations. Of interest, only up to 16% of the associated SNPs were located in protein-coding regions, whilst the majority of the variants were found to be in regulatory regions identified by transcription factor ChIPseq, DNAse I hypersensitivity and expression quantitative trait (eQTL) mapping. Maurano *et al*. [21] obtained similar results and further showed the cell type-specific localization of the regulatory marks overlapping with variants found to be associated with specific diseases in GWASs. This finding has also been confirmed for various histone marks [22] and eQTLs [23–25]. The enrichment of GWAS hits within regulatory regions of the genome, particularly in cells with disease-related functions, provides additional evidence for their direct involvement in disease.

**Figure 2:**
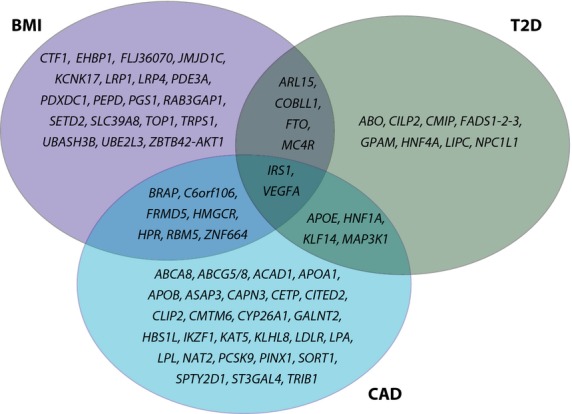
Overlap of genes associated with lipid levels and other cardiometabolic traits. The Venn diagram illustrates the overlap of genes found to be associated with plasma lipid levels by Willer et al. [27] and genes associated with body mass index (BMI), type 2 diabetes (T2D) and coronary artery disease (CAD).

Studies on the effects of SNPs on intermediate molecules such as RNA, proteins or lipids have been able to illustrate the functional consequences of disease-associated genetic variants, reviewed by van der Sijde *et al*. [26]. For example, plasma concentrations of lipoproteins and triglycerides are heritable but modifiable risk factors for CAD and T2D. In a genome-wide screen for common variants associated with plasma lipid levels in over 188 000 individuals, Willer *et al*. [27] demonstrated 157 significantly associated loci. The authors further investigated the associations between the 157 loci and body mass index (BMI), CAD, T2D and blood pressure and found that, although the functions of some genes remain unknown, many of the 40 genes associated with lipid levels and CAD play important roles in lipid metabolism, including *APOA1*, *APOE*, *HNF1A*, *LDLR*, *LPA*, *LPL*, *NAT2* and *SORT1*. They observed a smaller overlap between lipid levels and T2D-associated genes; the top hit amongst the 18 loci was *FTO*, one of the first genes to be associated with BMI and T2D [28,29]. Figure2 illustrates the overlap between the different genes associated with lipids and CAD, T2D and BMI.

The functional effects of many of the identified SNPs remain to be explored. For example, Musunuru *et al*., using functional studies of the rs12740374 SNP in *SORT1*, showed that the noncoding polymorphism creates a C/EBP transcription factor-binding site and thereby alters the expression of the SORT1 gene in hepatocytes [30]. The authors further demonstrated that Sort1 alters plasma low-density lipoprotein cholesterol (LDL-C) and very low-density lipoprotein (VLDL) particle levels by modulating hepatic VLDL secretion. Based on more specific analysis of T2D phenotypes, implicated genes have been clustered by risk alleles associated with the following: (i) primary effects on insulin sensitivity (*PPARG*, *KLF14*, *IRS1* and *GCKR*); (ii) reduced insulin secretion and fasting hyperglycaemia (*MTNR1B* and *GCK*); (iii) defects in insulin processing (*ARAP1*); and (iv) insulin processing and secretion without a detectable change in fasting glucose levels (*TCF7L2*, *SLC30A8*, *HHEX/IDE*, *CDKAL1* and *CDKN2A/2B*) [31]. Stratifying patients into different clusters based on risk alleles will result in better understanding of the disease pathways and eventually lead to an increase in therapy that is more tailored.

The possibility of translating the loci detected by GWASs into novel therapeutic targets is dependent on the identification of causal mutations and genes and their downstream effects on protein activity. Flannick *et al*. [32] speculated that loss-of-function mutations that protect against disease without adverse phenotypes would be amongst the most useful findings from human genetics, as such mutations may be directly translated into targets for therapy [33,34]. The authors sought to identify such targets by sequencing the exons of 115 genes near T2D association signals identified by GWASs and were able to find a rare nonsense variant (c.412C>T, p.Arg138*) in *SLC30A8* with protective effects against T2D. After further sequencing and genotyping of *SLC30A8* in 149 134 individuals, they found that heterozygosity for any of 12 identified protein-truncating variants was associated with a 65% reduced risk of T2D (odds ratio 0.34, *P* = 1.7 × 10^−6^) [32]. *SLC30A8* encodes a zinc transporter ZnT8 expressed solely in β-cells, and its overexpression has been shown to increase glucose-stimulated insulin secretion [35]. Sladek *et al*., who first discovered the association between *SLC30A8* and T2D, suggested that the finding may have dietary implications, including therapeutic approaches with zinc supplementation or pharmacological manipulation of zinc transport [36]. The recently identified loss-of-function protective mutations in *SLC30A8*, together with evidence that a high total zinc intake may attenuate the glucose-raising effect of another common SNP in this gene (rs11558471) [37], further indicate that *SLC30A8* could be an excellent target for pharmacological intervention.

Another field in which there have been rapid developments in GWASs and disease risk estimates is predisposition to breast cancer. The existence of a family history of breast cancer is one of the strongest risk factors [38], and the known pathogenic mutations in the *BRCA1* and *BRCA2* genes confer a life-time risk of breast cancer of 60–85% and 55–85%, respectively [39,40]. Recently, additional common alleles have been reported to be associated with increased breast cancer risk for *BRCA1* and *BRCA2* mutation carriers in large retrospective studies [40,41]. Although the effect associated with each of these SNPs is small, the combination of the alleles may be useful for the stratification of individuals into distinct risk categories [42]. Nevertheless, the combined frequency of *BRCA1* and *BRCA2* mutations in the general populations is nearly 0.5% [43,44]; thus, more common variants are more relevant for determining predisposition to breast cancer. GWASs and targeted genotyping projects have together identified 67 low-penetrance loci associated with susceptibility to breast cancer [45]. Together, findings indicate that 14% of familial risk of breast cancer can be explained by these common variants, with a further 20% by the loci with higher penetrance. By including more SNPs with less stringent criteria, Michailidou *et al*. [45] could increase the explained proportion from 14% to 28%, suggesting that increasing sample sizes will enable the identification of more risk loci with even smaller effect sizes.

## Disease risk estimates and patient stratification

As mentioned above, individual susceptibility alleles only confer a modest increase in disease risk; most odds ratio values are <1.5 or, with increased sample sizes, <1.2. Therefore, the predictive utility of genetic tests based on single risk alleles is poor. However, combining susceptibility alleles into polygenic risk scores has been shown to be more effective for risk prediction (see below). With the constant increase in identified disease susceptibility loci for different diseases, it is difficult to accurately assess how well the combined genetic risk scores perform, but a few generalizations can be made based on studies so far.

Tikkanen *et al*. [46] recently reported the clear utility of genetic risk scores for patient reclassification into low-, intermediate- and high-risk groups using 28 SNPs associated with coronary heart disease (CHD) in four Finnish cohorts (FINRISK). The SNPs with the largest effect sizes were located in *LPA*, *CDKN2A/B-ANRIL*, *CELSR2-PSRC1-SORT1*, *MRPS6*, *PPAP2B*, *MIA3* and *WDR12*. The authors noted that using the genetic risk scores to modify the traditional risk classification, 135 deaths could be prevented per 100 000 individuals over 14 years. This is presumably achieved by reclassifying individuals with intermediate-risk (10-year absolute risk of cardiovascular disease of 10–20%) into the high-risk (>20%) category, and expecting 32.5% of the individuals in the high-risk group to experience a CHD event based on the 14-year follow-up data, and that statin treatment would reduce the risk of an event by 20%. They also stated that screening for genetic risk scores would prevent 2.5 times more CHD events than randomly allocating statins to a comparable number of individuals in the intermediate-risk category using traditional classification and the same assumptions as above.

Based on the results from the latest breast cancer GWASs and assuming that all loci combine multiplicatively, the estimated relative risks for individuals in the 95th and 99th percentiles are 2.3 and 3.2, respectively, compared to the population average [45]. Although such estimations are limited for predicting breast cancer for any given individual, the risk scores would be useful for identifying high-risk individuals eligible for enrolment in screening and prevention programmes at an earlier age. The potential utility of polygenic risk stratification in the case of population-based screening for breast and prostate cancer has been reviewed by both Véron *et al*. [47] and Pashayan *et al*. [48]. The two groups of authors argued that risk and eligibility for screening according to age alone, as in the current UK national breast screening programme, is suboptimal as many women under the age for screening (<47 years) will develop breast cancer and most women above the age for screening (>73 years) will not. Alternatively, using 18 SNPs associated with breast cancer in a polygenic risk profile, personalized screening of women aged 35–79 years is expected to result in screening of 2% fewer women but yield the same number of potentially detectable cases as in the age-determined risk profile [49]. The situation is expected to improve even further by incorporating the more recently identified risk loci.

Similar progress has also been made in estimating combined genetic risk scores for other diseases, including rheumatoid arthritis [50], T2D [51,52], prostate cancer [49], age-related macular degeneration [53] and ischaemic stroke [54]. For many diseases, the genetic risk scores do not outperform the traditional factors considered in the clinic for risk assessment. However, with the increase in the number of known susceptibility genes, the estimates are becoming more precise, even compared to family history [55] and, most importantly, the genetic risk can be calculated before the manifestation of disease symptoms such as increased lipid levels, hypertension or the presence of autoantibodies. It is also important to note that the discriminatory power of polygenic risk scores may be population specific and may therefore need to be validated and reweighted in other populations.

Most complex diseases are caused by hundreds of different genetic polymorphisms and various environmental factors, and their interactions. The advantage of genetic risk factors is that they are present before disease onset and can therefore be used for stratification of individuals into risk categories for preventive screening. Early detection of disease progression is crucial for successful treatment of patients. Even though the genetic risks cannot be altered directly, other risk factors (such as BMI, lipids levels, smoking and alcohol consumption) for most diseases can be modified.

## Beyond GWAS – ‘omics’ data and rare variants

Similar to other biobanks, in addition to containing genetic information, the Estonian Biobank puts considerable effort into generating different levels of ‘omics’ data and updating health-related information for all participants. This enables the investigation of other factors associated with risk of a disease and the development of risk scores that take into account both genetic and nongenetic variables. The strength of using different levels of omics data and updated phenotype data was recently illustrated by researchers at the EGCUT [56]. By quantifying 106 candidate biomarkers in a random subset of over 9800 plasma samples from the Estonian Biobank, they identified four biomarkers that predicted the risk of all-cause mortality: alpha-1-acid glycoprotein, albumin, VLDL and citrate. One in five participants with a biomarker summary score within the highest percentile died during the first year of follow-up, indicating systemic reflections of frailty. The results were replicated in a population-based cohort from Finland (FINRISK 1997). Based on these and other findings, it is extremely important to work towards closer collaborations with clinicians and using the possibilities of recontacting subjects for further examination and potential treatment.

Cytogenetic testing using SNP microarray analysis for detection of chromosomal abnormalities in patients with developmental delay/intellectual disability, multiple congenital anomalies and autism spectrum disorders has been covered by the Estonian Health Insurance Fund since 2011. In a recent overview of 1191 patients analysed during the period 2009–2012, chromosomal abnormalities were identified in 25% of the patients; however, as the clinical significance of a large proportion (41%) of the findings remains unknown, clear and clinically relevant findings were reported for only 11% of the patients [57]. This is a twofold improvement compared to traditional cytogenetic methods, and chromosomal microarray analysis is now established as the first-line cytogenetic diagnostic test for detection of chromosomal abnormalities [57].

In parallel with studying common diseases, the EGCUT has worked closely with medical geneticists at Tartu University Hospital to implement whole-exome sequencing into clinical practice to improve patient care. Since the beginning of 2014, the Estonian Health Insurance Fund covers the costs of parent–offspring exome sequencing to identify the cause of rare diseases of unknown genetic aetiology. So far, 138 exomes have been sequenced (including 53 cases) with a diagnostic yield of 40%, which is comparable to that of other centres [58,59]. Actionable incidental findings are currently reported back to the clinician according to the recommendations of the American College of Medical Genetics and Genomics [60].

## Use of genetic data in health care

Several countries have recognized the need for and potential of personalized medicine and started national initiatives to facilitate the necessary research and development for its implementation. A summary of national and regional initiatives in personalized medicine is shown in Table2. In the USA, a broad spectrum of research, focusing on areas from medical genetics and genomics to translational and functional genomics and social and behavioural studies, is being conducted at the National Human Genome Research Institute [61]. In the UK, similar steps have been taken towards personalized health care [62]. The Public Health Genomics Foundation (PHG) [63] was established in 1997 to facilitate the integration of genetics and genomics into public health practice, with a focus on policy development for the UK National Health Service. With regard to the national healthcare system, the PHG Foundation report entitled *Public health in an era of genome-based and personalized medicine* concluded that (i) the focus should be on disease areas with significant population health impact, (ii) evidence on utility and cost-effectiveness of genomic approaches needs to be collected and (iii) this effort should be international.

**Table 2:** Examples of national and regional personalized medicine initiatives

Country	Brief description of the initiative
Australia	National Health and Medical Research Council (NHMRC) has prepared a framework for translating ‘omics-based’ discoveries into clinical care, including governing principles for clinical research, clinical practice and guidelines, data repositories and ethical/legal/social issues particularly related to return of results.
Austria	ONCOTYROL aims to facilitate advances in individualized cancer therapies, as well as the development and evaluation of diagnostic, prognostic and preventive tools.
	BioPersMed aims to identify specific biomarkers in endocrinology, cardiology and hepatology.
Belgium	Belgian Medical Genomics Initiative – a network to create an optimal national framework for clinical exome sequencing.
	Transformational Medical Research (TGO) – personalized medicine programme managed by the Agency for Innovation by Science and Technology.
	Biomina – a biomedical informatics research centre in Antwerp, created to facilitate translational medicine (including bioinformatics and medical informatics).
Canada	Genome Canada – partnered with the Canadian Institutes of Health to support the Large-Scale Applied Research Competition in Genomics and Personalized Health.
Denmark	Danes’ DNA catalogue is being created by the Danish Platform for Large-scale Sequencing and Bioinformatics through large-scale sequencing with the primary aim of developing vaccines against cancer.
England	Genomics England and 100 K Genome Project – mapping of 100 000 patients’ genomes through whole-genome sequencing (WGS) for identification of target variants for rare diseases, cancer and pathogens.
Finland	Finland Distinguished Professor Programme (FiDiPro) – promoting the use of personalized medicine in treatment of diseases focused on genome-scale cancer biology.
	Sequencing Initiative Suomi (SISu) aims to build tools for genomic medicine using whole-genome and whole-exome sequence and to make the data available for the research community.
France	Advanced Diagnostics for New therapeutic Approaches (ADNA) aims to develop more personalized therapeutics for infectious diseases, cancers and rare diseases.
Greece	The Genomic Medicine Alliance – current major projects include EuroPGx which genotypes pharmacogenomically relevant variants from samples in developing nations, and the pilot NextGenPGx project which aims to sequence whole genomes to create a database of the incidence of genetic disorders in three ethnic groups.
India	Human Genomic Initiatives and Genetic Epidemiology of Cancer plans the genetic cataloguing of ethnic groups, better prenatal care and the use of cancer genomics.
Japan	Implementation of Genomic Medicine Project (IGMP) aims to construct a network of disease-oriented and population-based biobanks, and to establish a medical genome centre which will establish optimized treatment through optimized diagnostics and prediction of drug responses using large-scale genomics.
Korea	Korean Genome and Epidemiology Study (KoGES) – large-scale population-based prospective cohort study which collects epidemiological data and WGS information.
	Korean Genome Analysis Project (KoGAP) has constructed the Korean reference genome.
Singapore	POLARIS programme implements genomic medicine in a city/state health system and aims to prove the clinical utility of genomic testing.
USA	Genomic Medicine Research Portfolio of the National Human Genome Research Institute (NHGRI) (an institute of the NIH) focuses on the advancement of human health through genomic research.
	The PharmGKB – a pharmacogenomics knowledge resource that encompasses clinical information including dosing guidelines and drug labels, potentially clinically actionable gene–drug associations and genotype–phenotype relationships.

NIH, National Institutes of Health. Sources: http://www.eurobioforum.eu/2028/observatory/and notes from Genomic Medicine Centers Meeting VI: Global Leaders in Genomic Medicine, 8–9 January 2014, National Academy of Sciences Building, Washington, DC. http://www.genome.gov/27555775.

In 2012, an international coalition of professional and patient advocacy groups, the European Alliance for Personalised Medicine (EAPM) [64], was founded with the goal of accelerating the development, delivery and uptake of personalized medicine and personalized health care and thereby improving patient care. The objectives of EAPM for 2013 and 2014 included creating incentives for personalized health care, by influencing related EU policies and developing reimbursement that would be favourable for the implementation of personalized medicine.

A global network, the Global Alliance for Genomics & Health, was established to accelerate progress in genomic research through sharing genomic and clinical data [65]. Partners from over 40 countries represent a variety of stakeholders including health care, research, disease advocacy, life sciences and information technology (IT) institutions. To achieve the goal of accelerating the progress in translating genomic research into healthcare practice through the combined implementation of genomic and clinical data, the working groups of the Global Alliance for Genomics & Health are working towards (i) facilitating interoperability in data representation, storage and analysis; (ii) harmonization of policies and best practices in genomic and health-related data sharing; and (iii) introducing interoperable standards for managing and sharing genomic and clinical data.

Whilst research into the role of common variants in common complex diseases continues, evidence regarding the use of genomic tests is also accumulating. Increasing numbers of genomic tests have been reported to provide sufficient evidence of clinical validity and utility and some of these have already been recommended for use in clinical practice by the Centers for Disease Control and Prevention in the USA [66]. The Charles Bronfman Institute for Personalized Medicine (IPM) is an example of a personalized medicine project launched to implement data-driven and genetics-based personalized health care [67]. The aim of this project is to use each patient's genetic information and clinical data for targeted, personalized care in real time, through the application of a personalized medicine system linked to EMRs. Additional evidence is gathered during routine clinical care, and electronic medical records are used to facilitate genomic research as proposed by the Electronic Medical Records and Genomics network (eMERGE) network [68].

In May 2013, the Prime Minister of Estonia acknowledged personalized health care as an appropriate strategy to manage the national increasing burden on health care of noncommunicable diseases, with an emphasis on disease prevention rather than treatment [69]. In 2014, the chairman of the management board of the Estonian Health Insurance Fund presented an action plan that included personalized approaches for disease prevention, emphasizing the need for appropriate funding models and research on outcomes including the behavioural and psychological impact of genetic risk predictions [70]. Most significantly, the newly formed Estonian Government included in its coalition agreement of 2014 the plan to develop personalized medicine based on modern gene technology [71].

The priority of the suggested predictive, preventive, personalized and participatory approach is health promotion for improving health care where there has been an increase in public health burden of noncommunicable diseases [72]. Whilst the personalized and predictive aspects of this approach rely on biomarkers for early diagnosing or for identification of at-risk patients, the preventive and participatory aspects rely on intervention at the primary care level where educating both patients and healthcare providers is crucial. The findings of a study of the expectations of Estonian primary care physicians regarding the use of genomic information in primary care practice suggested that there is an eagerness to apply genomic information in practice, as well as a willingness to improve their knowledge base in genetics and genomics [73]. However, there is a need for policies and guidelines, as well as clinical decision support, for determining where, when and how to use test results.

A major concern regarding personalized medicine is the lack of evidence of clinical utility and cost-effectiveness; validation is needed to ensure that the new approaches lead to positive health outcomes and are cost-effective. Policies and guidelines are necessary to regulate the level of evidence required before a new genetics- or genomics-based test is implemented. In addition to clinical outcomes, it is necessary to consider the psychosocial, ethical and legal implications of introducing genomic approaches into clinical care. Further challenges for the successful implementation of predictive, preventive, personalized and participatory approaches in healthcare practice include the need for (i) IT tools to integrate different sources and the combined analysis of patient data; (ii) user-friendly clinical decision tools to guide physicians in decision-making to introduce complex genomic-based approaches into clinical care; and (iii) funding schemes to promote and provide incentives for prevention rather than reactive treatment of disease.

## Future directions

**Figure 3:**
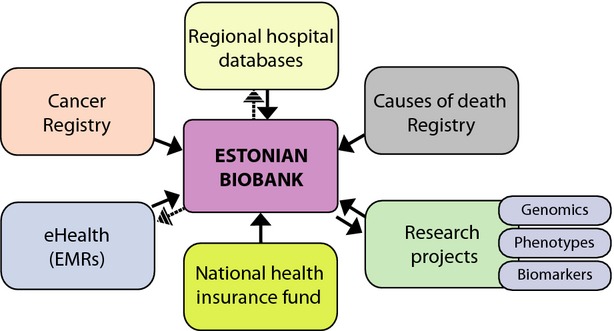
National registries and databases for enrichment of phenotype data in the Estonian Biobank. The schematic diagram illustrates the different layers of information available in the database of the Estonian Biobank, which is continually being updated by queries to the Estonian Causes of Death Registry, the Estonian Cancer Registry and the Digital Prescription Database of the Estonian Health Insurance Fund, as well as electronic medical records (EMRs) from the databases of the two major hospitals in Estonia. Data generated through research projects must be returned to the Biobank within 5 years of the original data release from the Biobank.

As discussed above, the EGCUT has established a biobank with over 52 000 extensively phenotyped participants and is a partner in various international research projects focusing on the identification of genes and environmental factors associated with increased risk of common diseases. Recent research efforts have shifted towards studying the underlying mechanisms of common complex diseases by including different levels of omics data, such as epigenetics, transcriptomics, proteomics and metabolomics. The phenotype data are also continually being updated by regular queries of national registries, including the Digital Prescription Database, as well as the databases of the two major hospitals in Estonia (covering approximately 75% of the population). A more detailed description of the phenotype data available in the registries and databases is provided by Leitsalu *et al*. (see their table 4) [13] and illustrated in Fig.3.

Estonia was the first country to implement a nationwide electronic health record system (eHealth) with full access to individual records for all citizens [74]. This system provides new opportunities for both citizens and healthcare providers in the era of personalized medicine. The EGCUT is currently working hard to build a system for implementing genomic information into the Estonian healthcare system. We believe that there is sufficient reliable information from different levels of omics data, including robust common and rare SNP risk alleles, plasma biomarkers and pharmacogenomics markers (reviewed by others in this issue of the *Journal of Internal Medicine*), to start the process of implementing personalized medicine. The disease risk predictions will improve as the process proceeds, and we learn from implementing personalized medicine based on what is currently known whilst maintaining a flexible IT system for incorporation of new findings.

The EGCUT has proposed a national plan for personalized medicine in Estonia, which is based on the initial sequencing of 5000 whole genomes to identify rare variants and haplotypes specific to the Estonian population. This will be followed by the design of a genotyping microarray using these haplotype-tagging variants and other polymorphisms known to be associated with disease risks. According to the plan, all samples in the Estonian Biobank will then be genotyped, the data will be analysed using automated risk estimation and decision support software, and disease risk and drug response prediction reports will be deposited into the e-Health system. Physicians will be trained to use the data in their everyday practice, and if this pilot phase is successful, the same test should be offered to all adult residents in Estonia. We hope to provide disease risk and medication response predictions directly to the healthcare providers by 2020.

We are convinced that the introduction of genomics together with currently used medical practice organized by user-friendly IT systems will lead to better screening programmes, earlier detection of diseases and better opportunities for treatment of patients. In short, genomics would provide an additional ‘instrument’ for physicians to diagnose and treat patients. Of note, genomic testing is the only tool that has true predictive value; compared to other medical tests, which usually only record the situation at the time that the test is performed when patients have developed disease symptoms, genomics will allow early screening and preventive methods for individuals at high risk of specific diseases, thereby reducing the burden on the healthcare system and health insurance funds.

## Funding

The establishment and maintenance of the Estonian Biobank has been supported by the Ministry of Social Affairs and the Ministry of Economic Affairs and Communications; the Estonian Biobank and the EGCUT have been supported by the Development Fund of the University of Tartu (grant SP1GVARENG), the Estonian Research Council (grant IUT20-60), the European Regional Development Fund to the Centre of Excellence in Genomics (EXCEGEN; grant 3.2.0304.11-0312) and through European Union's Seventh Framework Project (FP7) grants (278913, 306031, 313010, 245536, 269213, 212111 and 201413).

## Conflict of interest statement

No conflicts of interest to declare.
